# Antifungals influence the immune-related transcriptomic landscape of human monocytes after *Aspergillus fumigatus* infection

**DOI:** 10.1038/s41598-022-08738-4

**Published:** 2022-03-17

**Authors:** Benoît Henry, William Klement, Wajiha Gohir, Claire Aguilar, Shahid Husain

**Affiliations:** 1 Transplant Infectious Diseases, Ajmera Transplant Centre, Toronto General Hospital, University Health Network, 585 University Avenue, 11 PMB 138, Toronto, ON M5G 2N2 Canada; 2 Service des Maladies Infectieuses et Tropicales, Centre d’Infectiologie Necker Pasteur, Hôpital Universitaire Necker-Enfants Malades, Assistance Publique-Hôpitaux de Paris, Université de Paris, Paris, France; 3 Faculty of Computer Science, Dalhousie University, Halifax, NS Canada

**Keywords:** Cell biology, Fungal infection, Drug regulation, Infection

## Abstract

The heterogeneity of clinical responses to antifungals in aspergillosis is partially understood. We hypothesized that besides direct antifungal effects, these discrepancies may be related to different immunomodulatory profiles. Human THP-1 monocytes were coincubated in vitro with *Aspergillus fumigatus* and variable concentrations of voriconazole (0.5, 1 and 2 mg/l), caspofungin (1 and 2 mg/l), amphotericin B deoxycholate (0.25, 0.5 and 1 mg/l) and liposomal amphotericin B (1, 2 and 3 mg/l). After 6 h of coincubation, total cellular RNA was extracted, converted into cDNA, and transcription of 84 genes involved in antifungal immunity was measured through RT-qPCR. The presence of *A. fumigatus* was the main driver of the global immune-related transcriptomic response*.* After *Aspergillus* infection, thirty genes were upregulated, while 19 genes were downregulated. Discrepancies across antifungals were also evident; voriconazole-containing conditions showed similar reaction to natural infection, while the use of liposomal Amphotericin B significantly decreased the inflammatory response. Chemokines (notably CCL20 and CXCL2) and pro-inflammatory cytokines (IL1A, IL1B, IL23, G-CSF) exhibited the most pronounced differences across antifungals. Pattern recognition receptors and adaptor protein transcription were minimally affected. Protein–protein-interaction network analysis showed that IL23A played a dominant role in upregulated genes. Pathway enrichment analysis indicated that cytokine-cytokine receptor integration, TNF signaling pathways and Toll-like receptor pathways were highly involved. This exploratory study confirms the heterogeneous immunomodulatory role of antifungals. Overall, voriconazole appears to maintain an early pro-inflammatory response seen in natural infection. Assessment of immunomodulatory response with clinical response may provide a better rationale for differences observed across antifungals.

## Introduction

*Aspergillus fumigatus* is a ubiquitous filamentous fungus responsible for a broad range of human diseases, from allergic sinusitis or asthma to invasive and disseminated infection, through chronic, cavitary pneumonia^1^. *Aspergillus* is one of the four major fungal pathogens of clinical relevance worldwide^2^. As an example, the incidence of invasive aspergillosis is estimated to be 1.6/100,000 in Canada^3^. The majority of *Aspergillus*-associated diseases are due to *Aspergillus fumigatus,* which has expectedly been the most extensively studied species.

The principal inoculation route of *Aspergillus* is respiratory, and the organ mainly affected is hence the lung. In the lung, monocytes, alveolar macrophages, and alveolar epithelial cells represent the first line of antifungal defense. Any qualitative or quantitative alteration of these cellular populations can increase the risk of invasive aspergillosis. This is essentially encountered in neutropenia (mainly post-chemotherapy), myeloid hematologic malignancies, chronic granulomatous disease, and long-term use of immunosuppressives, as in organ transplantation^4^.

Treatment of aspergillosis mainly relies on systemic antifungals. The preferred molecule is voriconazole^5,6^, which interferes with the synthesis of membrane ergosterol in the fungus. This choice is mainly justified by the results of a seminal randomized controlled trial that demonstrated a global survival advantage favoring voriconazole compared with conventional amphotericin B^7^. Alternatives to voriconazole are isavuconazole and posaconazole, which were non-inferior to voriconazole in invasive mold infections^8^ in immunocompromised subjects and invasive aspergillosis^9^. Echinocandins, the beta D glucan synthesis inhibitors, are active against *Aspergillus fumigatus* but lack clinical evidence to be recommended as first-line therapy. They are, however, sometimes used in refractory cases or combination therapy^10^.

The differences of response across antifungal agents can be partly explained by heterogeneous modes of action or varied safety and pharmacokinetic profiles. However, these differences in the clinical efficacy of these antifungal drugs remain only partially understood. One of the explanations is the potential of differential effect on innate immune response by these antifungal drugs. Previous work with voriconazole found evidence of a modulation of transcription and translation of specific immune-related genes such as Toll-Like Receptors^11^. However, no comparative in vitro studies between different antifungal agents have been performed. We, therefore, hypothesized that antifungal-induced modulation of the innate immune response to *A. fumigatus* could vary between these antifungal agents. This study explored the differences in immune-related transcriptomic activity in human monocytic cell lines challenged with *A. fumigatus* and exposed to major systemic antifungals as a first exploratory step.

## Material and methods

### Human monocytes culture

Human THP1 monocytes (ATCC®TIB-202™) were maintained in culture in conventional medium (RPMI 1640 Glutamax (ThermoFisher Scientific, Waltham, MA, USA) supplemented with 10% Fetal Bovine Serum (ThermoFisher Scientific, Waltham, MA, USA) and 1% penicillin/streptomycin (Sigma-Aldrich, St Louis, MO, USA)). Concentration was adjusted not to exceed 10^6^ cells/mm^3^. Counting and viability assessments were made through a Vi-Cell XR® cell counter (Beckman Coulter, Indianapolis, IN, USA).

### Aspergillus fumigatus culture and hyphal growth

*Aspergillus fumigatus* (strain ATCC® 13073TM; NIH 5233) was cultivated on potato dextrose agar slants at 37 °C. To retrieve conidia, 5 ml of 0.05% Tween 80 was added to the slants, followed by vortexing for 30 s. The supernatant was then filtered through a 40 µm cell strainer (Fisherbrand, Waltham, MA, USA), and the conidial suspension was centrifuged at 4500 rpm for 10 min at room temperature. Conidia were washed twice with 0.05% Tween 80, then resuspended in 0.005% Tween 80 and counted with a hemocytometer.

We elected to co-incubate monocytes with *A. fumigatus* hyphae, given their more robust immunogenicity and more significant implication in the invasive stage of the disease. For hyphal growth, 10 million conidia were added to 50 ml of Sabouraud liquid medium (Sigma-Aldrich, St Louis, MO, USA) in a 250 ml Erlenmeyer flask partially closed with sterile cotton incubated at 37 °C, under continuous rotation (150 rpm). Germination of conidia and its homogeneity was confirmed through microscopic examination and followed by a wash step in sterile water.

### Antifungals

Liposomal amphotericin B was obtained from Gilead (Foster City, CA, USA). Voriconazole, caspofungin and amphotericin B deoxycholate were purchased from the University Health Network pharmacy department. Voriconazole, caspofungin and amphotericin B deoxycholate were diluted in DMSO at 1000 mg/l, 1000 mg/l, and 5000 mg/l, respectively, and stored at -80 °C. Liposomal amphotericin B was diluted in a culture medium at 1000 mg/l less than 12 h before coincubation and kept at 4 °C, protected from light.

### Co-incubation of THP1 cells and *A. fumigatus*

The following conditions were tested: THP1 cells; THP1 cells with *A. fumigatus* hyphae; THP1 cells with each antifungal, with and without *A. fumigatus* hyphae. Co-incubation was performed in 12-well non-tissue culture-treated plates. In each well, 10^6^ monocytes were co-incubated with 200 000 hyphae (multiplicity of infection: 5 hyphae for one monocyte), and antifungals were pre-diluted in 200 µl of culture medium. We used the following antifungals concentrations: for voriconazole, 0.5, 1 and 2 mg/l; for caspofungin, 1 and 2 mg/l; for amphotericin B deoxycholate 0.25, 0.5 and 1 mg/l; for liposomal amphotericin B, 1, 2 and 3 mg/l. These conditions were chosen based on the most frequently observed therapeutic drug concentrations of antifungals^5^. Each experimental condition was co-incubated for 6 h, at 37 °C, in duplicate. In all coincubations, THP1 cell viability was greater than 95%.

### Multiplex quantitative real-time PCR

At the end of coincubation, the 2 wells of each condition were merged, then spun, the cell pellet was resuspended in 700 µl of Qiazol (Qiagen, Hilden, Germany) and stored at − 80 °C. According to the manufacturer’s instructions, total RNA was extracted using the RNeasy mini kit® (Qiagen, Hilden, Germany). RNA concentration and purity were assessed through spectrophotometric measurement of the absorbance at 260 and 280 nm. In all conditions, absorbance 260/280 ratios were comprised between 1.91 and 2.05. According to the manufacturer's instructions, RNA was converted into cDNA using RT2 First Strand kit® (Qiagen, Hilden, Germany). Multiplex real-time qPCR was then conducted using the RT2 Profiler PCR Array Human Antifungal Response® (Qiagen, Hilden, Germany) according to the manufacturer’s instructions. Briefly, this multiplex assay evaluates the transcription of 84 selected genes based on their known involvement in antifungal immunity. Gene expression analysis data were normalized to 4 housekeeping genes (ACTB, B2M, GAPDH, HPRT1, RPLP0). Quality control procedures included PCR reproducibility, reverse transcription control, and elimination of genomic DNA contamination.

### Data analysis and statistical methods

Principal component analysis was performed using R software ggplot package (Integrated Development for R. RStudio, PBC, Boston, MA URL http://www.rstudio.com/). Graphs were generated with GraphPad Prism v7 (GraphPad Software, San Diego, CA, USA).

After normalizing to housekeeping genes, gene expression differentiation was established by at least 2 folds change up to or down and with statistically significant *p*-value < 0.05. Gene expression differentiation was calculated in two settings; (I) the first compared gene expressions in infected (but non-treated) THP1 monocytes to non-infected, non-treated THP1 cells, and (II) the second setting compared gene expressions in THP1 monocytes (treated by antifungals and also infected with *A. fumigatus*) to those gene expressions of infected but not treated THP1 controls. The latter comparison measured the ability of antifungals to reverse the gene expression response to infection in THP1 cells.

### Protein–protein interaction (PPI) networks and molecular pathways analysis

Significantly differentiated genes in response to infection (identified in setting I above) were used to construct two PPI networks. While genes that were upregulated in response to infection were used to construct the first PPI network, the second PPI network was based on those genes that were downregulated after infection. A third PPI network was also constructed from genes differentiated by 1 mg/l of liposomal amphotericin B to reverse the effect of infection in THP1 cells. We utilized the *StringDB* bioinformatic tool^12^ to construct and analyze all PPI networks. The PPI were restricted to over 90% confidence as calculated by *StringDB* system. The resulting PPI networks were then imported into the *Cytoscape* network integration tool^13^ for analysis.

Furthermore, we also performed pathway enrichment analysis for each PPI network to identify significantly enriched signaling pathways in each group of genes. This resulted in a statistically significant enrichment p-value less than 5.0 × 10^–16^ in all PPI networks we analyzed. The resulting signaling pathways were retrieved from the Kyoto Encyclopedia of Genes and Genomes database^14^ and the REACTOME pathway database^15^.

## Results

### Global immune-related transcriptomic response

**Figure 1:**
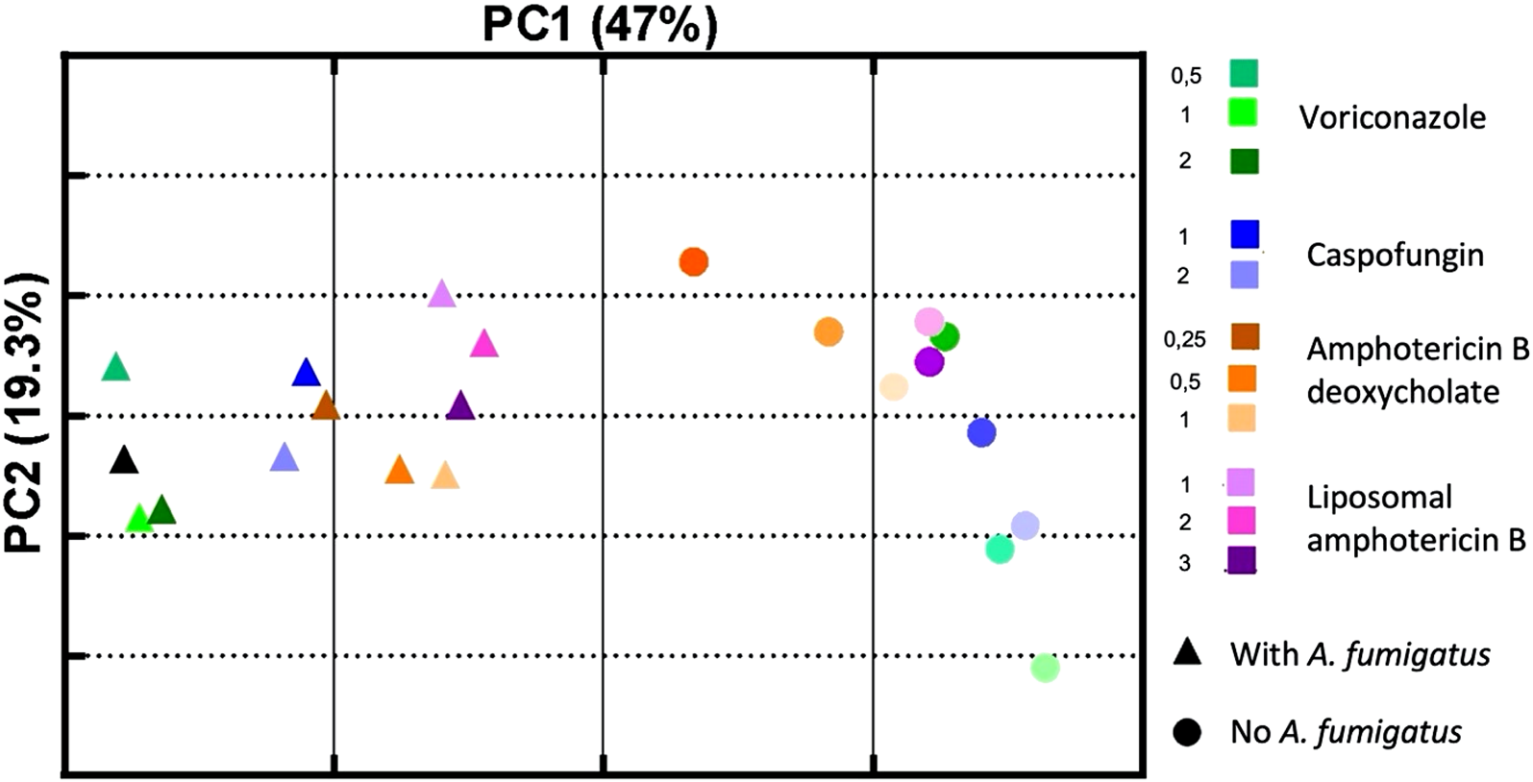
Principal component analysis of the global transcriptomic response for the 84 genes evaluated, across 22 experimental conditions (*Aspergillus fumigatus* isolatedly, 11 with antifungals alone, 11 with antifungals + *A. fumigatus*). The black triangle represents THP1 cells infected with *A. fumigatus*, without any antifungal.

Principal component analysis (PCA) was performed to analyze the global monocyte immune-related transcriptomic response to infection and the influence of antifungals on this response. We evaluated 84 genes by RT2 Profiler PCR Array, in 22 experimental conditions, resulting in PC1 (47% of variability) and PC2 (19% of variability) as shown in Fig. 1. The main driver of change in gene expression was the presence of *A. fumigatus.* Heterogeneity was observed across antifungals; conditions including voriconazole clustered independently of liposomal and deoxycholate amphotericin B, caspofungin conditions in an intermediate situation. This was more pronounced when *A. fumigatus* was included.

### Gene expression in natural infection and influence of antifungals

**Figure 2:**
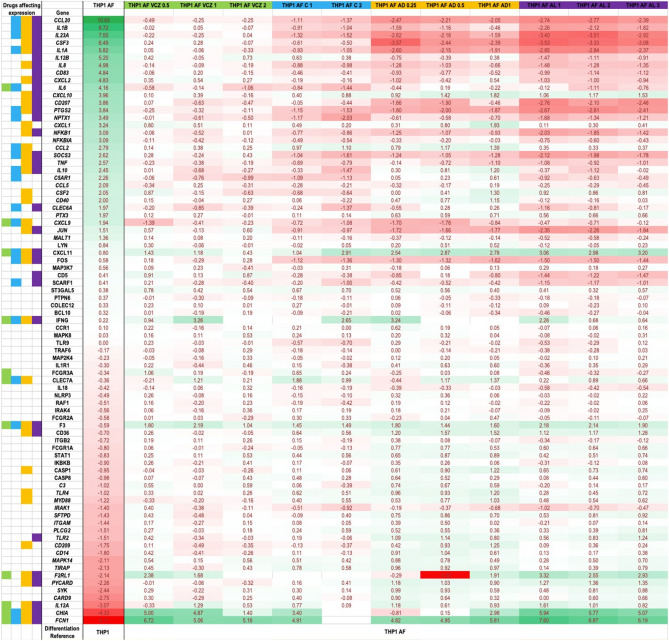
Log_2_(fold change) of gene expressions for 84 genes (rows) across 12 experimental conditions (columns). For column THP1 AF, the reference of differentiation is THP1 only. For the remaining columns, the reference is THP1 AF (infected, non-treated cells). Genes are sorted according to the intensity of their expression in THP1 AF column. A log2(fold change) $\ge$ 1 was of interest, and corresponding genes names are highlighted in green (up-regulation) and in red (down-regulation). Across antifungals-treated experimental conditions, fold regulations of interest are highlighted in corresponding colour on the left side (voriconazole: green; caspofungin: blue; amphotericin B deoxycholate: orange; liposomal amphotericin B: purple). AF: *Aspergillus fumigatus*; VCZ: voriconazole; C: caspofungin; AD: amphotericin B deoxycholate; AL: liposomal amphotericin B. Drug concentrations are in mg/l.

*Analysis I*: The heatmap in Fig. 2 shows the log_2_(fold change) of experimental conditions (columns) for evaluated genes (rows). While a positive entry depicts gene up-regulation, a negative entry represents down-regulation in gene expression, and further, log_2_(fold change) ≥ 1 indicates a fold change of at least two folds. THP1 AF column shows log_2_(fold change) for infected, non-treated THP1 cells relative to non-treated and non-infected THP1 controls, which represents the transcriptional response to *A. fumigatus* infection. In this case, 30 genes were upregulated and 19 genes were downregulated in response to infection. The upregulated genes included chemokines (7 genes: CCL20, CXCL2, CXCL10, CXCL1, CCL2, CCL5, CXCL9), pro-inflammatory cytokines (7 genes: IL1B, IL23A, IL1A, IL12B, IL8, IL6, TNF), and growth factors (2 genes: G-SCF and GM-CSF). Likewise, the down-regulated genes comprised receptors involved in innate recognition of the fungus (6 genes: C3, TLR4, SFTPD, ITGAM, TLR2, FCN1) and molecules implicated in signal transduction and acting downstream of innate immunity receptors (5 genes: MYD88, IRAK1, TIRAP, SYK, CARD9).

*Analysis II*: The remaining heatmap columns of Fig. 2 show log_2_(fold change) of gene expressions in infected and treated THP1 cells, compared to infected but non-treated THP1 cells labelled THP1 AF. With respect to the effect of antifungals doses, differences were observed within each molecule: among the genes whose expression was affected by antifungals, the proportion of genes that were significantly changed across all drug concentrations was 27%, 43%, 32% and 68% for voriconazole, caspofungin, amphotericin B deoxycholate and liposomal amphotericin B, respectively. However, these differences across doses were frequently minimal, as shown in Fig. 2.

Gene expression was affected by the presence of infection and subsequently, by the use of antifungals (Fig. 2; supplementary Figure S1). Overall, the 30 genes that were upregulated in response to natural infection (THP1 AF column) were minimally affected by the addition of voriconazole, whereas both formulations of amphotericin, and to a lesser extent caspofungin, down-regulated the majority of these genes (voriconazole: 2 genes down-regulated; caspofungin: 13 genes; amphotericin B deoxycholate: 15 genes; liposomal amphotericin B: 20 genes). These differences were less pronounced when considering the 19 genes that were down-regulated in response to natural infection; however, a proportion of genes was upregulated in treated conditions (voriconazole: 4 genes upregulated; caspofungin: 2 genes; amphotericin B deoxycholate: 10 genes; liposomal amphotericin B: 7 genes).

Regarding pattern recognition receptors, the expressions of Dectin 1 & 2, Collectin 12 and Mannose-Binding Lectin 2 was only marginally changed, with no apparent influence of antifungals. Toll-Like Receptors 2, 4 and 9 were overall mildly down-regulated in the presence of *A. fumigatus* and/or antifungals, with again no clear difference across molecules. A similar pattern was found regarding Ficolin 1 expression.

When addressing chemokine response, the monocyte-attractant CCL2 transcription was upregulated in the presence of the fungus; the addition of caspofungin and amphotericin B deoxycholate amplified this effect. CCL5 transcription, another monocyte attractant chemokine, was mildly enhanced in the presence of *A. fumigatus*, with no difference related to antifungals. CCL20 was highly upregulated in the presence of the fungus, and this persisted in association with voriconazole. This response was not sutained in the presence of other antifungals. Liposomal amphotericin B had the lowest values. The neutrophil-attractant chemokine CXCL8 was upregulated between 24 and 27-fold in the presence of voriconazole. This upregulation was less pronounced in the presence of caspofungin and amphotericin. A similar pattern was observed for CXCL2 (except for amphotericin B deoxycholate at 1 mg/l) but not for CXCL1. Transcription of CXCL9 was minimally affected across groups. CXCL10 & CXCL11 were upregulated in the presence of the fungus. This effect was amplified in the presence of both formulations of amphotericin B.

In terms of cytokine response, gene expression of interleukin 1α and β followed a similar pattern: when *A. fumigatus* was absent, changes were minimal but these cytokines were strongly upregulated in the presence of *A. fumigatus* (52 and 389 folds for IL1α and β, respectively). The presence of antifungals altered gene expressions variably ranging from being intense in the presence of voriconazole, less critical in the presence of caspofungin, and even less in the presence of the 2 formulations of amphotericin. Transcription of interleukin 6 was marginally changed in the absence of *A. fumigatus* (− 2.44 to 5.3 fold change) and upregulated in the presence of *A. fumigatus* and antifungals, with no apparent differences across molecules. Regarding interleukin 2, the only factor upregulating its transcription was liposomal amphotericin B. The expression of interleukin 18 was not significantly changed across experimental conditions. IL12-IL23-interferon γ axis was also explored: *A. fumigatus* decreased the expression of IL12A and increased the one of IL12B; liposomal amphotericin B induced a less intense transcription of IL12B compared with other antifungals. IL23 was upregulated in the presence of *A. fumigatus*; this response was identical in the presence of voriconazole but sharply decreased in other antifungals, especially liposomal amphotericin B. The transcription of interferon γ was unaffected.

Tumour Necrosis Factor (TNF)-α transcription was moderately increased by *A. fumigatus* (5.47 folds). TNF elevation was also observed in the presence of voriconazole and low concentrations of amphotericin B deoxycholate, but to a lesser extent in the presence of caspofungin and liposomal amphotericin B. The anti-inflammatory cytokine IL-10 was mildly upregulated in a dose-dependent manner in the presence of amphotericin B deoxycholate. The expression of IL-1R1 was unchanged across conditions. While GM-CSF transcription was marginally affected, G-CSF transcription was strongly upregulated (83 folds) by the presence of the fungus. This intense transcription was maintained in the presence of voriconazole, to a lesser extent in the presence of caspofungin but only mildly increased in the presence of the 2 formulations of amphotericin B.

### Molecular pathway analysis

**Figure 3:**
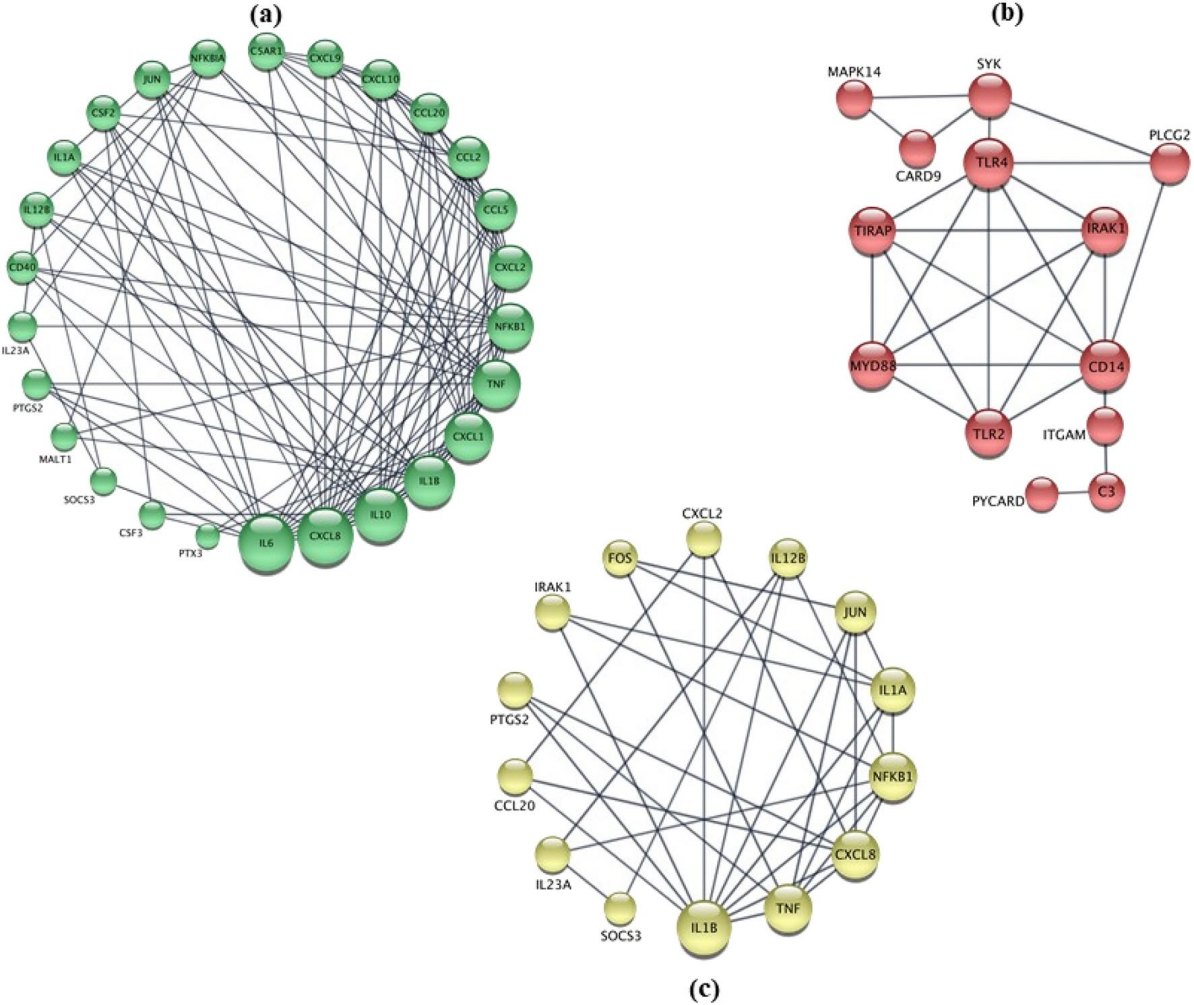
Protein–protein interation (PPI) networks of: (**A**) genes up-regulated by infection, (**B**) genes down-regulated by infection and (**C**) genes up-regulated by infection but also downregulated by applying 1.0 mg/l of AmphoB L (“THP1 AF AL1” column in Fig. 2).

The groups of upregulated and downregulated genes by more than 2 folds which are listed in Fig. 2 were used to construct two corresponding protein–protein interaction PPI networks shown in Figs. 3a,b (upregulated and downregulated respectively).

The network in Fig. 3a is based on the upregulated genes and included 30 proteins, 109 interactions, 8.4 average neighbours, and an enrichment *p*-value < 10^–16^. IL1B was most dominant interactor with most proteins except for IL23 A and SOC3. In contrast, the network in Fig. 3b was constructed from the downregulated and was smaller with only 13 proteins, 25 interactions, 3.8 average number of neighbours, and enrichment *p*-value also < 10^–16^. The PPI network based on upregulated genes was denser (more proteins), with higher connectivity between them (more interactions) than that constructed from the downregulated genes. This suggests that the response to infection in THP1 samples involved more upregulations than downregulation of genes.

Pathway enrichment analysis on the above PPI networks showed intense activities on various relevant signaling pathways. The PPI of upregulated genes included—among many—“*IL-17”* pathway (FDR < 10^–25^), “*Cytokines-cytokine receptor interaction*” pathways (FDR: 10^–24^), “*TNF*” pathway (FDR: 10^–24^), and “*Toll-like receptor*” signaling (FDR < 10^–18^). Conversely, the downregulated genes enriched substantially fewer signaling pathways, which were limited to “Cytokines signaling” (FDR: 10^–18^) and “Toll-like receptors” pathways (FDR: 10^–18^). The resulting pathways were confirmed in KEGG^14^ and REACTOME databases.

The heatmap in Fig. 2 shows log_2_(fold change) for various doses of antifungal drugs applied to infected THP1 samples. Clearly, applying 1.0 mg/l of AmphoB L (“THP1 AF AL1” column) produced the highest changes in genes upregulated by the infection. Thus, sorting the heatmap by “THP1 AF AL1” column identified genes subjected by the most fold change. From those, we identified 21 genes whose response to infection was reversed by the antifungal drug by > 2 folds. Further, a PPI network constructed from these genes is shown in Fig. 3C. This final PPI network offers insights into the proteins involved in the intense effect of the drug on infected THP1 samples. This network contained 21 proteins, 33 integrations, 4.7 average number of neighbours and enrichment p-value < 10^–16^. Its most significantly enriched pathways included “IL-17” signaling (FDR 10^–25^), “Cytokine-cytokine receptor” interactions (FDR < 10^–24^) and “Toll-like receptor pathways (FDR < 10^–18^).

## Discussion

This in vitro coincubation model involved human monocytes and *A. fumigatus* hyphae to study the effect of 4 different antifungals (3 being routinely used in treating *Aspergillus*-related diseases) on immune-related gene expression. We observed several differences between groups. Through PCA, our analysis demonstrated that although *A. fumigatus* drove most of the variability, significant differences were observed between antifungals, especially between voriconazole and amphotericin B. The response induced by voriconazole in the presence of *A. fumigatus* was similar to that induced by *A. fumigatus* alone.

Most significant gene expression changes were observed in chemokines, which supports their early involvement in the innate immune response mediated by monocytes. Neutrophils and monocyte-attractant (CCL2 and CXCL8) were intensely upregulated by *Aspergillus*, and this response was maintained to a certain extent in by voriconazole. The role of XCX chemokines in anti-*Aspergillus* immunity is well-documented^1^, and with respect to cytokines, a similar transcriptional profile was observed in IL1α, IL1β and IL23 consisting of a strong upregulation in case of natural infection, which was preserved by voriconazole but not by liposomal amphotericin B. These cytokines play an essential role in implementing Th1 response, a vital element of the adaptive anti-A. *fumigatus* response^16^. Interestingly, the transcription of few genes was enhanced by amphotericin B: CXCL10, IL10, IL2 (only by liposomal amphotericin B).

It is also noticeable that within each antifungal, there was a minimal effect of the dose used in our experiments, which may be related to the fact that we chose a relatively narrow range of concentrations, close to the usual therapeutic levels.

The transcriptional response was decreased for several pro-inflammatory genes in case of coincubation with liposomal amphotericin B. The ability of voriconazole to “preserve” immune response observed in *Aspergillus* infection can be seen as beneficial for the host—especially in the initial phase of disease—by limiting the spread of infection leading to better clinical outcomes, which is well-documented about the use of voriconazole.

Various studies investigated the potential immunomodulatory of antifungals on innate immune cells. To the best of our knowledge, this study is the first to simultaneously compare voriconazole, caspofungin, and the 2 most commonly used forms of amphotericin B. In the context of the literature, our experiments showed similar transcriptional responses regarding chemokines^11,17–19^ except CCL20 and CXCL2, whose expression was significantly more intense in our study. A similar pattern was observed for IL1β. The strong upregulation of CCL20 (and somewhat of CCL2 and IL12B) induced by voriconazole had not been observed by Simitsopoulou et al.^19^. Likewise, the minimal changes we observed in PRR transcription were consistent with Simitsopoulou et al.^19^. The transcriptional modifications induced by amphotericin B deoxycholate alone agree with previous works^19,20^. In the presence of *A. fumigatus*, the strong transcriptions of CCL2, CCL20, IL10 and IL1β that we observed were not previously noted^19^. The transcriptional effect of liposomal amphotericin B in the presence of *A. fumigatus* had not been studied. Yet, similar experiments were conducted by Turtinen et al*.*^20^, using amphotericin B lipid complex: the overall minimal effect of these formulations on chemokines transcription was also found here with liposomal amphotericin B (except CXCL10, significantly upregulated with Amphotericin B Lipid Complex). The observed moderate upregulation of IFN gamma transcription observed by Turtinen et al*.*^20^ was not observed here. It is plausible that discrepancies in experimental protocols (duration of incubation, concentrations of antifungals, multiplicity of infection, type of *Aspergillus* strain, morphologic form of *Aspergillus* used) played a role in this heterogeneity. The pro-inflammatory effect of amphotericin B and its lipid formulations has essentially been observed in neutrophils, not in monocytes^21^.

We acknowledge that PPI Networks and pathways analysis presented may be subject to selection bias attributed to the selective nature of gene panel used in this study. However, these genes have been shown associated with immune response to *Aspergillus* infection^1^. Moreover, we applied a highly stringent confidence threshold (90%) of interactions when constructing PPI networks to counter potential noise.

This study has limitations; an in vitro coincubation between monocytes and *Aspergillus* fails to account for in vivo complexity of infection at the scale of an organ and cellular populations at stake. For example, caspofungin and voriconazole appear to modulate the antifungal activity of NK cells^22^, which could not be evaluated here. This study explored gene expressions in innate immune cells; further studies can characterize differences in corresponding protein expression to deconvolute mechanisms involved. Although THP-1 monocytes form a well-recognized cell line that recapitulates functions of classical monocytes^23^, our study did not investigate the effect of *Aspergillus* and antifungals on transcription of monocytes whose function was altered by immunosuppression. Although this is a common practice, the presence of antibiotics in the culture medium may have modified the baseline transcriptomic response of THP-1 cells. Additionally, the use of primary human monocytes, instead of THP-1 cells, would have allowed an even more clinically relevant in vitro model. However, comparison of our gene expression results with previous works coincubating primary human monocytes with *Aspergillus* hyphae showed minimal discrepancies, as detailed in Supplementary Table S1. We also elected to use a well-characterized *Aspergillus fumigatus* strain, which eliminated potential, and clinically relevant, variations across *Aspergillus fumigatus* strains in terms of virulence^24^. The dynamic mechanisms of innate immune response to fungi might not have been captured after 6 h of co-incubation. However, 6 h was widely used in preceding studies, including a recent comprehensive transcriptomic work evaluating dendritic cells^25^; it was shown that transcriptional changes in stimulated THP-1 cells were detectable up to 6 h after stimulation^26^. Our experimental conditions were comparable to those used in 2 prominent studies^18,19^. This study used the hyphal form of *A. fumigatus*, representing the fungus's invasive form. However, the natural history of *Aspergillus* infections begins with penetrating the conidia of the airways; it is well acknowledged that conidia triggers a very different response in the innate immune cells present in the alveolar spaces and the human airways. Certainly, replicating this work after coincubation with conidia, and germinating conidia, would allow for a more comprehensive perspective of antifungals influence on anti-*Aspergillus* response. This is particularly relevant in high-risk immunocompromised hosts on antifungal prophylaxis, such as allogeneic stem cell transplant recipients or lung transplant recipients. In this case, conidia would enter in contact with innate cells that have been pre-exposed to antifungals.

In conclusion, this exploratory study offered evidence of interesting differences across major antifungals on the immune-related transcriptome of human monocytes, especially between voriconazole and amphotericin B. The chemokines and pro-inflammatory cytokines transcriptions were particularly affected. The fact that voriconazole preserved the response that resembled the one induced by natural *A. fumigatus* infection may account, at least partly, for the more favorable outcomes observed with using this molecule.

## Supplementary Information

Supplementary Information.
