# Supplementary material for: Antifungals influence the immune-related transcriptomic landscape of human monocytes after Aspergillus fumigatus infection
Source: Sci Rep. 2022 Mar 17;12:4581. doi: 10.1038/s41598-022-08738-4 (PMC8931103; doi:10.1038/s41598-022-08738-4)
Supplement: Supplementary file 1 — Supplementary Information. [file 41598_2022_8738_MOESM1_ESM.docx]

**Supplementary Information File**

**Antifungals influence the immune-related transcriptomic landscape of human monocytes after Aspergillus fumigatus infection**

**Authors**

Benoît Henry, William Klement, Wajiha Gohir, Claire Aguilar, Shahid Husain


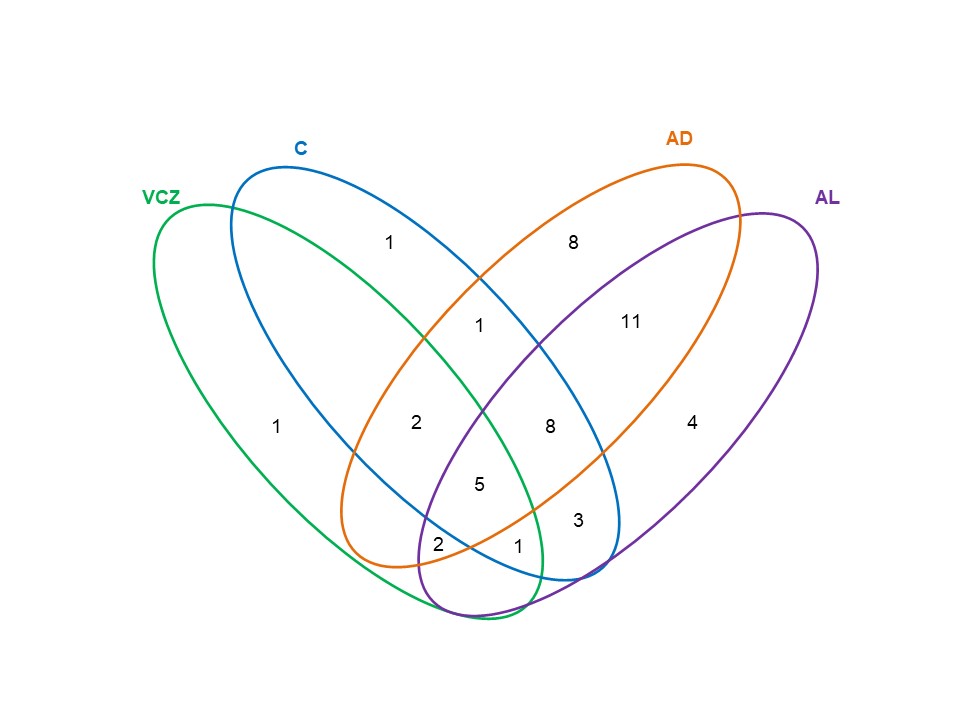


**Supplementary Figure S1: Venn diagram of the number of genes with a differential expression of more than 1 or less than -1 Log2(fold regulation), according to the drug used**. A gene was considered affected in case of differential expression with at least one of the drug concentrations used. VCZ, voriconazole; C, caspofungin; AD, amphotericin B deoxycholate; AL, liposomal amphotericin B.

| **Gene** | **Present study** | **Wang 2001**^1^ | **Loeffler 2009**^2^ | **Klassert 2017**^3^ |
| --- | --- | --- | --- | --- |
| *CCL20* |  |  |  |  |
| *IL-6* |  |  |  |  |
| *MALT1* |  |  |  |  |
| *TNF* |  |  |  |  |
| *CXCL1* |  |  |  |  |
| *IL1R1* |  |  |  |  |
| *PTX3* |  |  |  |  |
| *CASP1* |  |  |  |  |
| *CD14* |  |  |  |  |
| *SYK* |  |  |  |  |
| *PYCARD* |  |  |  |  |
| *IL8* |  |  |  |  |
| *IL1B* |  |  |  |  |
| *CCL2* |  |  |  |  |

**Supplementary Table S1** : **comparison of gene expression data from the present study (second column) and previously published literature of transcriptomic response of human primary monocytes when coincubacted with *Aspergillu*s hyphae**. Green boxes indicate increased expression, red boxes indicate decreased expression, and grey boxes indicate no significant change in gene expression. White boxes : no data.

**References :**

1. Wang, J. E. *et al.* Involvement of CD14 and toll-like receptors in activation of human monocytes by Aspergillus fumigatus hyphae. *Infect. Immun.* **69**, 2402–2406 (2001).

2. Loeffler, J. *et al.* Interaction analyses of human monocytes co-cultured with different forms of Aspergillus fumigatus. *J. Med. Microbiol.* **58**, 49–58 (2009).

3. Klassert, T. E. *et al.* Differential Effects of Vitamins A and D on the Transcriptional Landscape of Human Monocytes during Infection. *Sci. Rep.* **7**, 40599 (2017).
